# Does the Development of the Insurance Industry Promote the Purchase of Rural Commercial Health Insurance?

**DOI:** 10.3389/fpubh.2021.695121

**Published:** 2021-07-22

**Authors:** Cong Li, Si-Fan Wang, Xi-Hua Liu, Li Wang

**Affiliations:** ^1^School of Economics, Qingdao University, Qingdao, China; ^2^School of Economics, Ocean University of China, Qingdao, China; ^3^Medical College, Liaocheng University, Liaocheng, China

**Keywords:** commercial insurance, insurance purchasing behavior, China, DEA-CCR model, CFPS

## Abstract

Deepening the reform of insurance companies and improving commercial insurance protection capabilities become issues important to national strategy. They involve improving China's multi-tiered social security system to analyze the deep-seated reasons impacting the purchasing behavior of commercial health insurance for rural residents in China. Using the DEA-CCR model, this paper evaluates the development of China's insurance industry, inspects the impact of insurance industry development on purchasing behavior of rural commercial health insurance based on the data of tracking survey projects from China's household, and carries out empirical analysis. The research result shows that the development of the insurance industry has obviously promoted the purchase behavior of commercial health insurance for rural residents. This research has significant practical value on protection and promotion of production and life quality of rural residents, which will also provide beneficial reference on the formulation and implementation of future operation strategy in China's commercial health insurance companies.

## Introduction

Currently, China's economic development is shifting from an export-oriented to a domestic-demand-driven type, while the key to expand domestic demand is to effectively improve household consumption. In 2019, the growth rate of China's per capita disposal income reached 8.9%, and the growth of per capita consumption expenditure during the same time was 8.6%, which remained almost the same, while the growth of savings per capita reduced to 8.7%. ^①^This demonstrates that, while Chinese residents' income level was increasing rapidly and the consumer initiative was improving, residents' traditional saving concepts were changing to some degree. Nowadays, the coverage rate of endowment and medical insurance in Chinese society is over 90%, and the rural social insurance system has entered the phase of normative development, which has played a positive role in increasing farmers' income, promoting farmers' consumption and the harmony of rural families, and ensuring that the elderly in rural areas will be looked after. The social welfare in rural families is realized (1, 2). However, due to restraining factors such as low affordability levels, lack in policy coverage, and large regional differences in social security transfer payments, there are still some restrictions in the protection function of rural social insurance system toward rural residents and families (3, 4). It is not possible to realize 100% of loss compensation or 100% of income replacement by only relying on a social insurance system (5–7). Obviously, it is difficult helping rural residents and families to achieve more comprehensive and higher protection by only relying on social insurance system dominated by government (8, 9). The 14th Five-Year Plan clearly highlighted the reform and improvement of the social insurance system, the improvement of the insurance system, the promotion of social insurance transfer and continuation, and the improvement of a unified national social insurance public service platform. Therefore, building a multi-tiered security system is a necessary measure of “helping those most in need.” As an important supplementary form, commercial health insurance could play a part in risk protection, lever magnifying, credit financing, preventing and reducing damages, and main linkage, in order to satisfy the diversified protection needs among people, but with its increasing participation degree in ensuing people's livelihood, commercial health insurance has obviously become an important aspect of the multi-tiered security system (10–13).

It is necessary to emphasize that, on one hand, the acceleration of the popularization of commercial health insurance in rural areas will help improve the safeguard function of commercial health insurance in rural areas, which is significant in protecting rural residents' production and life. In recent years, the No. 1 central documents issued by the State Council all mentioned acceleration in development of rural insurance industry, establishment and improvement of rural medical insurance system, active implementation of protection measures in various insurance, and meeting the basic medical needs of rural residents (14, 15). As an important part of improving the social security system, the popularization of commercial health insurance combines safety security and risk investment, and protects life and property rights of the insured as far as possible. Therefore, as the areas with close attention in building a moderately prosperous society in all aspects, rural areas should facilitate the development of commercial health insurance business, and improve the relatively backward situation of insurance company service. On the other hand, in the 40 years since resumption of insurance business, China's insurance business has been flourishing^②^, and more and more residents tend to transfer traditional bank deposit properties into insurance assets, which brings more opportunity to the insurance companies. As of April 2020, there have been 232 insurance companies in different areas in China^③^, among which 97 were life insurance companies, taking up an important position in the insurance industry. Especially in the beginning of 2020, when the epidemic was spreading, insurance services represented by life insurance companies actively responded to the epidemic prevention work guidance of China's Banking Regulatory Commission, simplifying the claim service channel and maintaining clients' demands to the greatest extent, so as to provide strong support in the implementation of anti-epidemic work (16, 17). However, due to the dual impacts of internal industry and external environment, and some of the insurance companies paying too much emphasis on increasing the market share, the operation efficiency was showing a downward trend, which seriously influenced the companies' operational management level and the residents' purchasing behaviors of commercial health insurance. Additionally, the popularization of commercial health insurance in rural residents and families will further reduce the burden of caring for the elderly and increase the capability of consumption and risk taking, so as to further improve the life quality of rural residents (18, 19).

However, in the current situation of China's economy, the degree of participation of commercial health insurance in China's rural residents is not very optimistic, compared to that of urban residents^④^. One of the reasons is that rural residents have relatively weak risk awareness due to the limitations of funding and education. Another reason might be that insurance companies have not done enough publicity in rural areas, so that rural residents have limited understanding of insurance business. With the increase in rural residents' income and demand for protection, the factors influencing rural residents to purchase insurance are increasing. Therefore, whether the development of the insurance industry will influence the purchasing behavior of commercial health insurance among rural residents deserves further study.

## Literature Review and Research Hypothesis

As to the research on residents' purchasing behavior of commercial health insurance, there were many scholars at home and abroad conducting research from the aspects of individual condition, social relations, and current social situation. In terms of individual condition, Pu et al. (10) discovered that the education level of individuals would influence the purchase of family life insurance, meaning that the higher the education level was, the more likely an individual was to purchase insurance (20–22). Wang et al. (23) analyzed the relationship between psychological factors (individual value, risk attitude, and faith) and purchasing behaviors (24, 25). According to the research, there was an obvious negative correlation between risk attitude and life insurance purchase, while there were no such obvious relationship between individual value and faith and life insurance purchase. In terms of social relations, Wang et al. (12) and Liu et al. (13) reached the conclusion that social behaviors, including social interaction behavior and social trust degree, would influence insurance needs, through the study of participation degree of residents' commercial health insurance (26, 27). Wang et al. (23) also mentioned that an important factor influencing insurance demands were the trust degree of Chinese residents (28). If the individual had a relatively low trust degree toward insurance, it would directly influence their insurance purchase demands. Zong et al. (15) put forward that there existed a substitutional relationship between the two (29). In terms of social capital and social stratum, Wu (17) argued that the two had a positive influence on insurance purchasing (30). Taking Hong Kong as the research area, Keung et al. (18) investigated the factors influencing the external issues, such as how policy variables relating to society influence insurance purchase (31). According to research by Xiong et al. (19), Qiu (20), and Zhou et al. (21), purchase of commercial health insurance would have a reverse impact on the allocation and selection of household asset portfolio (32–34). Jia et al. (22) also used the CFPS database to conduct empirical analysis, showing that the out migration for work of rural residents would significantly decrease the families' purchase desire for commercial health insurance (35). In addition, Zhang et al. (24) and Zhang (25) studied the factors influencing the intent of purchasing life insurance and its premium (36, 37). The result showed factors such as people's worries about the future, economic conditions, knowledge of life insurance, chronic diseases, and preference for adventures would have obvious and positive influences on intent of purchasing life insurance. Christophe et al. (26) used cross-sectional data of the European Survey of Health, Aging, and Retirement to estimate the determinants of possibilities of purchasing long-term care insurance in France, wherein risk-taking behaviors and disabilities' experience played an important part (38, 39). By establishing health care need models, Ding and Zhu (27) evaluated the influence of medical care utilization on purchase of public health insurance (40). Based on the above theoretical research, this paper proposed the below research hypothesis:

**Hypothesis 1:** The development of the insurance industry is influenced by factors such as the number of employees in the insurance industry, fixed-asset investment, premium income, an compensation expenses.

The number of employees in the insurance industry can reflect the scale of the industry to some degree, and also reflect the labor capital input condition of insurance companies. Generally, the more employees there are, the larger the scale is, which means providing more convenient service for the applicants, and it is easier for applicants to get insurance interest protection, forming a positive cycle (41). The higher the performance obtained through man-power input channel, the higher the operation efficiency is. While from another aspect of capital input, fixed-asset investment can reflect the physical capital input status of an insurance company. The higher the fixed-asset investment is, the larger the premium scale and market share of the insurance company is (42). Correspondingly, the higher the company's operation efficiency is, the better the development situation of the insurance industry is. From the aspect of insurance company sales, premium income and compensation expenses are the two influencing factors affecting the development of the insurance industry. Premium income can directly measure the gross output of the insurance company. More output means a stabler operation of the insurance company, so as to promote the improvement of the operation efficiency. The compensation expenses, as one of the important measures of risk tolerance ability of insurance companies, can reflect the companies' operation condition to some degree (43). It is necessary to combine compensation expenses and premium income to comprehensively evaluate the development condition of insurance industry.

**Hypothesis 2:** The development of the insurance industry has a significant impact on the purchasing behavior of commercial health insurance among rural residents.

It is known from hypothesis 1 that the development of the insurance industry is influenced by various factors, among which promoting the development of the insurance industry from the perspective of human resource is mainly realized by enhancing sales level, business proficiency, working ability, and emergency reaction ability of insurance company staff. However, rural residents have limited knowledge and acceptance of insurance. Therefore, it is important to promote development of the insurance industry via the above mentioned measures so as to provide more convenient and efficient services to rural residents and bring more protection to farmers (44, 45). In this way, it is easier for rural residents to better accept insurance products and promote purchase of commercial health insurance (46, 47).

Based on the above literature review, most of the scholars had generally the same evaluation idea of insurance industry development, which used methods including DEA, SFA, and Malmquist exponential model to measure and analyze the development of the insurance industry, based on different research subjects (including various types of insurance companies such as life insurance companies, property insurance companies, and agricultural insurance companies). As to the study on residents' purchasing behavior of commercial health insurance, scholars had less research on the differences on consumption concepts between urban and rural residents, with the background of China's urban-rural dualization. Additionally, in terms of the study of influence factors of insurance purchasing behavior, most of the scholars favored the analysis on factors about individual or family situation, while there was less research in the macro aspects, with no literature referring to the related research as to whether the development of the insurance industry had influence on insurance purchasing behavior.

In view of this, this paper takes the development of the insurance industry and purchasing behavior of the commercial health insurance among rural residents as research objects and deeply investigates the influence of the development of the insurance industry on rural residents' insurance purchasing behavior. Considering the availability of data, this paper proposes to use the DEA-CCR model to conduct efficiency evaluation on life insurance companies in China's 25 provinces and cities^⑤^ in the years of 2013 and 2015, then uses data of Chinese household tracking survey projects CFPS in the years of 2014 and 2016, establishing the regression models of the development of the insurance industry and purchasing behavior of commercial health insurance, inspecting the influence of the development of the insurance industry on purchasing behavior among rural residents. Its significance is to promote the development of the insurance industry and provide rural residents with more reliable insurance protection.

The contribution margin of this paper mainly lies in the following three aspects: (1) The existing research mainly takes urban residents as research objects, without considering the heterogeneity in insurance purchasing behavior between urban and rural residents with the background of China's urban-rural dualization. Therefore, this paper takes rural residents as a research object, discussing and analyzing the influence of the development of the insurance industry on purchasing behavior of commercial health insurance among rural residents, and providing a theoretical basis for expanding rural commercial health insurance business; (2) The existing research mainly discusses influence on insurance purchasing behavior from the micro perspective of resident individuals or resident families, with only a few scholars conducting discussion from the perspective of the insurance industry. Therefore, this paper focuses on the influence of the development of the insurance industry on insurance purchasing behavior among rural residents, analyzing whether the insurance industry would influence the residents' purchasing behavior of commercial health insurance, which provides theoretical support for policy formulation and implementation, with good practice value.

The structure of the rest of the paper is as follows: The third part is the data source and variable setting, the fourth part is the empirical analysis, and the fifth part is the research conclusion and enlightenment.

## Data Sources and Specification of Variables

### Data Sources

The data adopted in this paper is from Chinese household tracking survey projects CFPS between the years of 2014 and 2016. The information data and research objects of this survey are highly reliable and representative. In order to increase the scientificity of the research, the sample data in this paper is processed as below: First, the population with non-rural registered permanent residence are removed; Second, due to the large size of sample, a lot of the data are missing, which are also removed; finally, in order to prevent the error influence on statistical result due to abnormal value, the last 1% of the data are winsorized, which leads to 17,874 final valid sample data.

### Specification of Variables

#### Variable Definition

In the analysis of factors of purchasing insurance among rural residents, it is mainly considering the purchasing situation of commercial health insurance among rural residents. There is a survey statistic in the questionnaire asking about “commercial insurance expenses in the past 12 months,” the result of which could reflect the residents' purchasing situation of commercial health insurance to some degree. Therefore, this paper sets the definition of explained variables as whether the family purchased commercial health insurance, which means that if the family's expense on purchasing commercial health insurance is <0, the variable is 0, otherwise the variable is 1.

Given that the influence factor researched in this paper is mainly about development conditions of the insurance industry, the operation efficiency of insurance companies is set as core explained variables. However, there is no survey relating insurance efficiency value in the database. According to the questionnaire setting in the database, it is discovered that there is division of provinces in the samples involved in the survey. Combining the insurance companies' efficiency in different provinces measured in Chapter II, the corresponding insurance companies are assigned for different sample data in this chapter according to province code.

In order to exclude the influence of other factors, making the analysis result more scientific, this paper also includes control variables into regression analysis. Many scholars have proved that population variables could influence the individual participation degree of commercial health insurance, the variables including age, gender, marriage, self-evaluation of health, and diseases. Referring to analytic demonstration of control variables of Qin et al. (28), this paper finally chooses age, gender, marriage, education level, diseases, self-evaluation of health, neighborhood relations, and household size as control variables.

Based on the above analysis, the variable description of this paper is as shown in Table 1.

**Table 1:** Description and assignment of the variables.

**Variable**		**Variable's name**	**Assignment**
Dependent variable	Purchase commercial health insurance	Insurances	Not purchased = 0, purchased = 1
Core independent variable	Operating efficiency of insurance companies	Efficiency	Efficiency value of insurance company
Control variable	Age	Age	The actual age of the rural population
	Age squared	Age2	The actual age squared of the rural population
	Gender	Gender	Female = 0, male = 1
	Marriage	Marriage	Unmarried = 0, married = 1
	Education level	Education	Whether it's high school/technical secondary school or above: No = 0, yes = 1
	Disease	Diseases	Are there any chronic diseases within 6 months: No = 0, yes = 1
	Health self-assessment	Health	From 1 to 5, the higher the better
	Neighborhood relations	Social	From 1 to 5, the higher the better
	Family size	Family	Number of rural households

#### Index Construction

Under the premise of complete data collection, this paper finally chooses insurance companies in 25 provinces of China to be decision-making samples of efficiency evaluation, selecting 2013 and 2015 as the evaluation section of operation efficiency. In terms of choosing index of insurance efficiency evaluation, this paper refers to the research and practice of Liu (29), Guo et al. (30), and Hong et al. (48), and selects value-added method as the evaluation basis of input and output index (47, 48, 49). At the same time, combining the research hypothesis 1 proposed as above, this paper finally chooses four indexes, namely insurance employee number, fixed asset investment, premium income, and compensation expenses, to conduct efficiency evaluation on insurance companies, wherein the insurance employee number and fixed asset investment are two input indexes, and premium income and compensation expenses are two output indexes. The index selection and descriptive statistics is shown in Table 2.

**Table 2:** Index selection and descriptive statistics.

**Index variable**	**Index description**	**2013**				**2015**			
		**Min**	**Max**	**Mean**	**Std**	**Min**	**Max**	**Mean**	**Std**
Invest-in index	Number of insurance practitioners	1	26.1	124	6.4	1	45	21.4	6.4
	Investment in fixed assets	2.1	159.4	47.4	37.9	4.5	150.8	50.5	37.1
Output index	Premium income	93	1243	424	281	124	1502	588	355
	Payouts	28	266	106	61	40	375	165	98

Based on data envelopment analysis DEA-CCR model, this paper calculates the operation efficiency of insurance companies in China's 25 provinces in the years of 2013 and 2015, and conducts regional classification according to regional classification standards^⑥^, as shown in Table 3.

**Table 3:** Comprehensive scale technical efficiency value of insurance company.

**Area**	**Serial number**	**Province**	**2013**	**2015**	**Mean**
Eastern area	1	Beijing	0.612	0.586	0.599
	2	Tianjin	0.729	1.000	0.865
	3	Hebei	0.839	0.571	0.705
	4	Shanghai	0.815	0.770	0.793
	5	Jiangsu	1.000	1.000	1.000
	6	Zhejiang	1.000	0.742	0.871
	7	Fujian	0.750	0.632	0.691
	8	Shandong	0.858	0.573	0.716
	9	Guangdong	1.000	0.816	0.908
		Mean	0.845	0.743	0.794
Central area	10	Shanxi	1.000	1.000	1.000
	11	Anhui	0.579	0.600	0.590
	12	Jiangxi	0.756	0.677	0.717
	13	Henan	1.000	1.000	1.000
	14	Hubei	0.524	0.447	0.486
	15	Hunan	0.628	0.495	0.562
		Mean	0.748	0.703	0.726
Western area	16	Guangxi	0.471	0.406	0.439
	17	Chongqing	1.000	0.974	0.987
	18	Sichuan	0.877	0.701	0.789
	19	Guizhou	0.694	0.332	0.513
	20	Yunnan	0.615	0.648	0.632
	21	Shanxi	0.687	0.556	0.622
	22	Gansu	0.636	0.579	0.608
		Mean	0.711	0.599	0.656
North-eastern area	23	Liaoning	0.737	0.528	0.633
	24	Jilin	0.652	0.665	0.659
	25	Heilongjiang	0.691	0.618	0.655
		Mean	0.693	0.603	0.649
		Total mean	0.766	0.677	0.721

On the whole, in 2013 and 2015, the average technical efficiency of the comprehensive scale of Chinese insurance companies were not high, at 76.6 and 67.7%, respectively; this shows that there might be resource waste to some degree in the operation management of China's insurance industry, with large space of improvement in efficiency of insurance companies, meaning that it is necessary to improve the development of the insurance industry and make up for the deficiency. Compared to 2013, the technical efficiency of the comprehensive scale lowered entirely. One reason might be that the insurance companies in some of the provinces were influenced by external environment or other factors, so that the improvement of industry technical level was not obvious. On the other hand, this was also a wake-up call to the insurance companies with low efficient value, encouraging the companies to enhance business management, improve operation efficiency, and realize improvement of technical efficiency on a comprehensive scale. It is worth mentioning that the technical efficiency of comprehensive scale of Shanxi, Jiangsu, and Henan in the years of 2013 and 2015 all remained as 1, which meant that the development of insurance industry in these provinces were steady.

From the view of the operation efficiency of insurance companies in 2013, the technical efficiency of the comprehensive scale in six provinces were 1, which meant that the resource utilization and operation management of insurance companies in these provinces were good. Additionally, there were 11 provinces with efficiency lower than 70%, among which, the operation efficiency of insurance companies in Guangxi was the lowest, at only 47.1%, which meant that these insurance companies might have serious problems in technique or management. From the view of efficiency in 2016, the operation efficiency of the nation's insurance companies was 67.7%, which slightly dropped compared with 2013. The number of provinces with technical efficiency on a comprehensive scale as 1 descended to 4 from 11, while the efficiency in most of the provinces had not reached the ideal status, and there was still a large gap compared to the valid efficiency. It is necessary for each insurance company to analyze the problems in a timely manner, make corresponding configurations and adjustments, improve operation efficiency, and stimulate the further development of the insurance industry.

From the regional perspective, probably because of the geographical advantages, the east and middle areas have a relatively high technical efficiency of comprehensive scale, which are 79.4 and 72.6%, respectively. While comparing with other regions, the economy and population development of west and northeast areas are in an inferior position, with relatively low technical efficiency of comprehensive scale, 65.6 and 64.9%, respectively. To sum up, the operation efficiency is high in the north and low in the south, and low in the west and high in the east. Therefore, in the west and northeast areas, it is necessary to focus on the problems, draw on strengths of each area to improve its own weakness, and pay attention to promote the operation efficiency of insurance companies, so as to stimulate the orderly and steady development of the insurance industry.

## Empirical Analysis

### Designation of Models

Based on sample data features, this paper chooses to use the Probit model to inspect whether the development of the insurance industry would have influence on the purchasing behavior of commercial health insurance among rural residents. The variable explained as *Y* in the Probit model is a variable 0/1, which is *f*(*X*) = *P*(*Y* = 1). It is a non-linear model following normal distribution. In other words, the probability of *Y* = 1 is a function of *X*, wherein *f*(*X*) follows the standard normal distribution.

The Probit model established in this paper is as function (1):

$$\begin{array}{l} Pr\left(insurance\;=1\right)\;=\;f\left(a\;+\;b*X_{i}\;+\;r*controls\;+\;\varepsilon_{i}\right) \end{array}$$

Among this, *i* means different provinces. *insurance* shows whether commercial health insurance is purchased in the survey samples, the variables being 0 or 1; *X*_*i*_ is the core explained variable; *controls* is the above mentioned control variable; and ε_*i*_ is the stochastic error term following normal distribution.

### Benchmark Regression

The benchmark regression result of the development of the insurance industry on the purchasing behavior of commercial health insurance of rural residents is shown in Table 4, among which, result (1) is the OLS regression only containing core explained variables, result (2) is the OLS regression containing core explained variables and all control variables, result (3) is Probit regression only containing core explained variables, and result (4) is the Probit regression containing core explained variables and all control variables. According to the empirical result, the efficiency index of insurance companies is positive, and is significant under the level 1%, which means that in the overall environment of insurance industry's healthy development, improving operation efficiency of insurance companies, namely improving the development level of insurance industry, could promote the insurance purchase of rural residents, which initially verifies the research hypothesis 2 in this paper. This also means that insurance companies should enhance companies' business capability, staff working capability, and emergency reaction capability, and stimulate the development of insurance industry in all aspects. As rural residents have limited knowledge on insurance, if more convenient and efficient services could be provided, rural residents will naturally have better acceptance and cognition of insurance, which will bring better protection in benefits of rural residents.

**Table 4:** Basic regression results.

**Variable's name**	**OLS regression**		**Probit regression**	
	**(1)**	**(2)**	**(3)**	**(4)**
Efficiency of insurance companies	0.0307^***^	0.0342^***^	0.2268^***^	0.2691^***^
	(0.0120)	(0.0127)	(0.0892)	(0.0988)
Age		0.0058^***^		0.0368^***^
		(0.0007)		(0.0559)
Age squared		−0.0000^***^		−0.0003^***^
		(0.0000)		(0.0005)
Gender		−0.0122^***^		−0.0935^***^
		(0.0040)		(0.3158)
Marriage		0.0278^***^		0.1893^***^
		(0.0055)		(0.4111)
Education level		0.0044		0.0287
		(0.0066)		(0.0501)
Disease		0.0138^***^		0.1271^***^
		(0.0057)		(0.0489)
Health self-assessment		−0.0013		−0.0107
		(0.0017)		(0.0139)
Neighborhood relations		−0.0076^***^		−0.0591^***^
		(0.0023)		(0.0183)
Family size		0.0031^***^		0.0245^***^
		(0.0010)		(0.0083)
Regional fixed effect	No	Yes	No	Yes
*N*	17,874	17,874	17,874	17,874

*The coefficients in the table are marginal coefficients, standard errors are in parentheses;*

*** *indicates significance at the 1% level*.

Additionally, the regression coefficient of the influence factors, such as age, marriage, diseases, and household size, is also positive, which means that they all have positive influence on insurance purchase of rural residents. As the analysis of insurance purchase factors in most of the literature shows, the older the age, the greater the attention to health, so the greater the need of insurance. Moreover, the square regression coefficient of age is negative, which means that there is an inverted U-shaped relationship between age and insurance purchase, and as the age increases, the margin impact shows a downward trend. According to the regression result, married residents are more likely to purchase insurance, and there is a similar influence of household size on insurance purchasing. The reason for the similar result of the two may originate from the influence of the family environment. Married residents and those with a large household size need to undertake more obligations than others, so the protection of insurance is not only targeted to individuals but also families. In the meantime, as a measurement of health condition, diseases have obvious influence in insurance purchasing. In order to get more favorable medical treatment and better protection of life and health, residents with diseases tend to purchase more insurance than healthy residents, especially life insurance.

The regression indexes of gender and neighborhood relationship are significant but negative, which means that they have obvious negative influence in insurance purchase of rural residents. From the regression result, the purchase behavior of females is more than that of males, and many scholars have had the same conclusion. Neighborhood relationship is a reflection of social skills, which has a negative correlation with insurance purchase in the research of this paper. Food neighborhood means high social skills, which leads to less prominent insurance needs; otherwise, there will be more contradiction and conflicts, and there is no doubt that the life and property security would undergo a bigger threat, which will increase the possibility of insurance purchasing, using insurance to protect one's own safety.

Finally, it can be seen from Table 4 that the regression indexes of education level and self-evaluation of health are positive and negative, respectively, but without a significant result. This shows that the two have, respectively, positive and negative correlation with insurance purchase, but the influence degree is not high. The above empirical results accord with the conclusions of related literature, which shows that the higher the education level is, the higher the possibility of insurance purchasing; the better the health condition is, the lower the insurance purchasing desire is.

### Robustness Test

In order to test the accuracy of benchmark regression, the related variables data was processed again. First, the data of variables being explained and control variables is replaced by the statistic result of CFPS2016, then the data of core explained variables is changed into operation efficiency of insurance companies in different provinces in 2015, so as to measure the development of the insurance industry. According to the new data statistic, the data is put into benchmark regression model to conduct a robustness test. The regression model and benchmark used by the robustness test is the same, wherein results (1) and (3) show the regression only containing core explained variables, and results (2) and (4) show the regression containing core explained variables and all control variables. The regression result is as shown in Table 5.

**Table 5:** Robustness test regression results.

**Variable's name**	**OLS Regression**		**Probit Regression**	
	**(1)**	**(2)**	**(3)**	**(4)**
Efficiency of insurance companies	0.0466***	0.0289***	0.2210***	0.2380***
	(0.0177)	(0.0111)	(0.0844)	(0.0865)
Age		0.0050***		0.0328***
		(0.0009)		(0.0066)
Age squared		−0.0000***		−0.0002***
		(0.0000)		(0.0006)
Gender		−0.0085*		−0.0706***
		(0.0042)		(0.0325)
Marriage		0.0433***		−0.2630***
		(0.0066)		(0.0464)
Education level		0.0006		0.0080*
		(0.0118)		(0.0660)
Disease		0.0315***		0.2937***
		(0.0060)		(0.0624)
Health self-assessment		−0.0026		−0.2142
		(0.0019)		(0.0145)
Neighborhood relations		−0.1050***		−0.7848***
		(0.0026)		(0.0194)
Family size		0.0023***		0.0156***
		(0.0010)		(0.0082)
Regional fixed effect	No	Yes	No	Yes
*N*	17,874	17,874	17,874	17,874

The coefficients in the table are marginal coefficients, standard errors are in parentheses; *** and * indicates significance at the 1 and 10% level.

From the regression result, the efficient index of insurance companies is significantly positive, meaning that the operation efficiency of insurance companies have obvious stimulation function in insurance purchase of rural residents, which is the same with the above content, and again verifies research hypothesis 2. This proves robustness in the benchmark regression model.

## Research Conclusions and Enlightenment

This paper investigates the operation efficiency of insurance companies in China's 25 provinces in the years of 2013 and 2015, analyzes in details the influence of the development of insurance industry on purchasing behavior of commercial health insurance among rural residents, and reaches three main conclusions: (1) By studying the influence factors of insurance purchasing among rural residents, it is discovered that the development of the insurance industry has significant facilitation of purchasing behavior of commercial health insurance among rural residents. The higher the operation efficiency of an insurance company is, the better the insurance industry development is, and the greater the purchasing power of rural residents is. (2) After analyzing the development of the Chinese insurance industry, it is discovered that there are differences in the development of insurance industry in different regions. In the meantime, the research shows that there are situations such as resource waste and low technique level in some insurance companies. The staff business capability, working capability, and emergency reaction capability need to be improved.

Based on the above research conclusions and the actual situation in China, the following enlightenment is obtained in this paper: First, at the same time as facilitating social security construction, the country could provide more preferential policy and guidance toward operation management of insurance companies and help the industry to develop orderly and steadily. In the meantime, it is important to focus on the insurance needs of rural populations, popularize insurance knowledge, and enhance grassroots ability to obtain cutting-edge information and judgement of risk estimation, so as to ensure that insurance guarantees are fully implemented. Second, it is necessary for insurance companies to continuously improve operation efficiency and promote the better development of insurance industry. On one hand, it can achieve resource allocation with maximum efficiency by taking full advantage of company resources and equitable distribution, and correcting the issue of resource waste; on the other hand, it can develop online insurance and other new forms of business on the basis of current business mode by improving company's innovation level, so as to increase premium income and cultivate company's competitiveness. At the same time, it is necessary to formulate targeting policy measures to facilitate healthy development of the insurance industry, according to the actual development situation of different areas, and combining the demand conditions of insurance among local residents.

## Data Availability Statement

The original contributions presented in the study are included in the article/supplementary material, further inquiries can be directed to the corresponding author/s.

## Author Contributions

CL: conceptualization, methodology, and software. S-FW: data curation and writing—original draft preparation. X-HL: visualization and investigation. LW: writing—reviewing and editing. All authors contributed to the article and approved the submitted version.

## Conflict of Interest

The authors declare that the research was conducted in the absence of any commercial or financial relationships that could be construed as a potential conflict of interest.

## References

[1] Garden LA Grace MF. X-efficiency in the US life insurance industry. J Bank Fin. (1993) 17:497–510. 10.1016/0378-4266(93)90048-I

[2] Chen C Cheng Z Jiang P Sun M Zhang Q Lv J. Effect of the new maternity insurance scheme on medical expenditures for caesarean delivery in Wuxi, China: a retrospective pre/post-reform case study. Front Med. (2016). 10:473–80. 10.1007/s11684-016-0479-2 27896623

[3] Ghimire R. Efficiency of Nepalese Life Insurance Companies Using DEA Approach. Social Science Electronic Publishing (2017) 7:2349–25. 10.16962/EAPJFRM/

[4] Cummins JD Tennyson S Weiss MA. Consolidation and efficiency in the US life insurance industry. J Bank Fin. (1999) 23:325–57. 10.1016/S0378-4266(98)00089-2

[5] Biener C Eling M Wirfs JH. The determinants of efficiency and productivity in the Swiss Insurance Industry. Eur J Operat Res. (2015) 248:117–48. 10.2139/ssrn.2577301

[6] Zhao X. An empirical analysis on the market behavior and market performance of Chinese insurance companies. Econ Rev. (2003) 24:118–21. 10.2991/icmess-18.2018.197

[7] Huang W. An empirical study on the efficiency of Chinese insurance institutions based on SFA method. Nankai Econ Stud. (2006) 18:104–15. 10.1016/j.geoderma.2019.07.035

[8] Sun R Feng TW. The efficiency of insurance companies operating agricultural insurance and its influencing factors–Based on SBM model and DEA window analysis method. Insurance Stud. (2016) 33:43–53. 10.3233/JIFS-17482

[9] Su CW Sun TZ. Does institutional quality and remittances inflow crowd-in private investment to avoid Dutch Disease? A case for emerging seven (E7) economies. Shabbir Ahmad Nawazish Mirza Resour Policy. (2021) 22:24–32. 10.1016/j.resourpol.2021.102111

[10] Pu CY Pan XJ. Research on the mechanism of behavioral financial stocks of insurance consumption promoting economic growth. Econ Res J. (2012) 38:139–47. 10.1016/j.jacceco.2010.10.003

[11] Suang ST Chee CL. A preliminary study on the relationship between psychographic factors and the purchase of life insurance. Int J Manage Stud. (2017) 24:1–22.

[12] Wang HQ Liu ZH Zhang YZ Luo ZJ. Integration of current identity-based district-varied health insurance schemes in China: implications and challenges. Front Med. (2012) 6:79–84. 10.1007/s11684-012-0179-5 22460451

[13] Liu H Wang J. Research on Chinese residents' medical insurance purchase behavior–based on the perspective of commercial health insurance. China Econ Q. (2012) 28:1525–48. 10.21203/rs.3.rs-31005/v1

[14] Wang KH Su CW. Does high crude oil dependence influence Chinese military expenditure decision-making? Energy Strateg Rev. (2021) 35:21–31. 10.1016/j.esr.2021.100653

[15] Zong QQ. Social pension insurance and China's household risky financial asset investment–evidence from China household finance survey (CHFS). J Fin Res. (2015) 12:99–114. 10.1016/j.labeco.2021.101978

[16] Wang HK Xiong D Mirza N Shao X Yue X. Does geopolitical risk uncertainty strengthen or depress cash holdings of oil enterprises? Evidence from China. Pac Basin Fin J. (2021) 66:101516. 10.1016/j.pacfin.2021.101516

[17] Wu YF. Social class, social capital, and commercial insurance purchase behavior of urban and rural residents in my country–based on the survey data of CGSS2015. China Soft Sci. (2018) 23:56–66. 10.1080/17516234.2016.1167413

[18] Keung LK Frankie Y Pui LY. Impact of the concerns over exogenous factors towards the insurance purchase: a study in Hong Kong insurance market. In: 7th International Joint Conference on Computational Sciences and Optimization. Beijing (2014). 10.1109/CSO.2014.162

[19] Xiong J Zhang SQ Zhao H Zeng XH. Optimal proportional reinsurance and investment problem with jump-diffusion risk process under effect of inside information. Front Math China. (2014) 9:965–82. 10.1007/s11464-014-0403-5

[20] Qiu JP. Precautionary saving and health insurance: a portfolio choice perspective. Front Econ China. (2016) 11:232–64. 10.3868/s060-005-016-0015-0

[21] Zhou Q Basu K Yuan Y. Does health insurance coverage influence household financial portfolios? A case study in urban China. Front Econ China. (2017) 12:94–112. 10.3868/s060-006-017-0005-7

[22] Jia HW Wang XQ. Will the behavior of rural residents going out to work promote the purchase of family commercial insurance?–Empirical research based on CFPS data. J Lanzhou Univers Fin Econ. (2019) 12:73–82. (In Chinese).

[23] Wang KH Liu L Lobont OR Nicoleta-Claudia M. Energy consumption and health insurance premiums in China's provinces: evidence from asymmetric panel causality test. Front Public Health. (2021) 9:658863. 10.3389/fpubh.2021.658863 33996730 PMC8116495

[24] Zhang XM Zhang YX Qiu HG. An empirical study of the key factors affecting consumers' purchase decision on life insurance. In: 4th International Conference on Service Systems and Service Management. Chengdu (2007). 10.1109/ICSSSM.2007.4280111

[25] Zhang CC. Chronic diseases, labor supply and medical expenditure at older age: evidence from China. Front Econ China. (2013) 8:233–59. 10.3868/s060-002-013-0012-7

[26] Christophe C Nolwenn R. Empirical evidence on long-term care insurance purchase in France. Geneva Pap Risk Insur Issues Pract. (2008) 33:645–58. 10.1057/gpp.2008.30

[27] Ding JH Zhu ML. A theoretical investigation of the reformed public health insurance in urban China. Front Econ China. (2009) 4:1673–3444. 10.1007/s11459-009-0001-8

[28] Qin F Wang WC He JC. The impact of financial knowledge on commercial insurance participation–an empirical analysis of data from the China Household Finance Survey (CHFS). J Fin Res. (2016) 23:143–58. 10.2139/ssrn.3393747

[29] Liu YX. An empirical analysis of the impact of internet insurance development on the operating efficiency of insurance companies. Insur Stud. (2015) 26:104–16. 10.1109/ICSSSM.2017.7996267

[30] Guo W Jing P Sun WJ. The source of potential for improvement in operating efficiency of property insurance companies in my country-based on the non-parametric common frontier analysis framework. Insur Stud. (2015) 93–103. 10.1016/j.ecoenv.2015.109540

[31] Charnes A Cooper WW Rhodes E. Measuring the efficiency of decision making units. Eur J Operat Res. (1978) 2:429–44. 10.1016/0377-2217(78)90138-8

[32] Esteban E Albiac J. Groundwater and ecosystems damages: questioning the Gisser-Sanchez effect. Ecol Econ. (2011) 70:2062–9. 10.1016/j.ecolecon.2011.06.004

[33] Biancardi M Maddalena L. Competition and cooperation in the exploitation of the groundwater resource. Decis Econ Fin. (2018) 18:219–37. 10.1007/s10203-018-0217-0

[34] Biancardi M Maddalena L. Groundwater management and agriculture. In: International Scientific Conference on IT, Tourism, Economics, Management and Agriculture. (Iraq) (2019). 10.31410/itema.2018.992

[35] Crettez B Hayek N Zaccour G. Non-deceptive counterfeiting and consumer welfare: a differential game approach. Ann Int Soc Dyn Games. (2020) 17:253–96. 10.1007/978-3-030-56534-3_11

[36] Xepapadeas A. Regulation and evolution of compliance in common pool resources. Scand J Econ. (2005) 107:583–99. 10.1111/j.1467-9442.2005.00424.x

[37] Negri DH. The common property aquifer as a differential game. Water Resour Res. (1989) 25:9–15. 10.1029/WR025i001p00009

[38] Bennett D Gosnell H Lurie S Duncan S. Utility engagement with payments for watershed services in the United States. Ecosyst Serv. (2014) 8:56–64. 10.1016/j.ecoser.2014.02.001

[39] Klapper L Panos GA. Financial literacy and retirement planning: the Russian case. J Pension Econ Fin. (2011). 10:599–618. 10.1017/S1474747211000503

[40] Lusardi A Mitchell OS. Financial literacy around the world: an overview. J Pension Econ Fin. (2011) 10:497–508. 10.1017/S1474747211000448 28553190 PMC5445931

[41] Su CW Qin M Zhang XL Tao R Umar M. Should bitcoin be held under the U.S. Partisan conflict. Technol Econ Dev Econ. (2021) 27:1–19. 10.3846/tede.2021.14058

[42] Burness HS Brill T. The role for policy in common pool groundwater use. Resour Energy Econ. (2001) 23:19–40. 10.1016/S0928-7655(00)00029-4

[43] Wang KH Adebayo TS Lobon OR Claudia MN. Fiscal decentralization, political stability and resources curse hypothesis: a case of fiscal decentralized economies. Resour Policy. (2021) 72:102071. 10.1016/j.resourpol.2021.102071

[44] Loehman ET. Pricing for water conservation with cost recovery. Water Resourc Res. (2008) 44:W08450. 10.1029/2008WR006866

[45] Robinson J. Future subjunctive: backcasting as social learning. Futures. (2003) 35:839–56. 10.1016/S0016-3287(03)00039-9

[46] Tao R Su CW Xiao YD Dai K Khalid F. Robo advisors, algorithmic trading and investment management: wonders of fourth industrial revolution in financial markets. Technol Forecast Soc Change. (2021) 163:23–36. 10.1016/j.techfore.2020.120421

[47] Su CW Qin M Rizvi SKA Umar M. Bank competition in China: a blessing or a curse for financial system. Econ Res. (2020) 34:1–21. 10.1080/1331677X.2020.1820361

[48] Hong JY Xu J. Research on the impact of internet insurance development on the efficiency of insurance companies—based on the two-step analysis of DEA-Tobit model. East China Economic Management, (2019) 33:103–10. (In Chinese).

